# Cervical cancer benefits from trabectedin combination with the β-blocker propranolol: *in vitro* and *ex vivo* evaluations in patient-derived organoids

**DOI:** 10.3389/fcell.2023.1178316

**Published:** 2023-06-13

**Authors:** Roberta Di Fonte, Sabino Strippoli, Marianna Garofoli, Gennaro Cormio, Simona Serratì, Vera Loizzi, Rossella Fasano, Francesca Arezzo, Mariateresa Volpicella, Afshin Derakhshani, Michele Guida, Letizia Porcelli, Amalia Azzariti

**Affiliations:** ^1^ IRCCS Istituto Tumori “Giovanni Paolo II”, Bari, Italy; ^2^ Unit of Obstetrics and Gynecology, Department of Interdisciplinary Medicine, Policlinico Hospital, “Aldo Moro” University of Bari, Bari, Italy; ^3^ Department of Biosciences, Biotechnologies and Environment, University of Bari, Bari, Italy; ^4^ Department of Microbiology, Immunology and Infectious Diseases, Cumming School of Medicine, University of Calgary, Calgary, AB, Canada

**Keywords:** cervical cancer, trabectedin, propranolol, ovarian cancer, patient-derived organoids

## Abstract

**Background:** Cervical cancer (CC) is characterized by genomic alterations in DNA repair genes, which could favor treatment with agents causing DNA double-strand breaks (DSBs), such as trabectedin. Hence, we evaluated the capability of trabectedin to inhibit CC viability and used ovarian cancer (OC) models as a reference. Since chronic stress may promote gynecological cancer and may hinder the efficacy of therapy, we investigated the potential of targeting β-adrenergic receptors with propranolol to enhance trabectedin efficacy and change tumor immunogenicity.

**Methods:** OC cell lines, Caov-3 and SK-OV-3, CC cell lines, HeLa and OV2008, and patient-derived organoids were used as study models. MTT and 3D cell viability assays were used for drug(s) IC_50_ determination. The analysis of apoptosis, JC-1 mitochondrial membrane depolarization, cell cycle, and protein expression was performed by flow cytometry. Cell target modulation analyses were carried out by gene expression, Western blotting, immunofluorescence, and immunocytochemistry.

**Results:** Trabectedin reduced the proliferation of both CC and OC cell lines and notably of CC patient-derived organoids. Mechanistically, trabectedin caused DNA DSBs and S-phase cell cycle arrest. Despite DNA DSBs, cells failed the formation of nuclear RAD51 foci and underwent apoptosis. Under norepinephrine stimulation, propranolol enhanced trabectedin efficacy, further inducing apoptosis through the involvement of mitochondria, Erk1/2 activation, and the increase of inducible COX-2. Notably, trabectedin and propranolol affected the expression of PD1 in both CC and OC cell lines.

**Conclusion:** Overall, our results show that CC is responsive to trabectedin and provide translational evidence that could benefit CC treatment options. Our study pointed out that combined treatment offset trabectedin resistance caused by β-adrenergic receptor activation in both ovarian and cervical cancer models.

## 1 Introduction

The vast majority of cervical cancer (CC) is caused by human papillomavirus (HPV), so it is currently preventable through a combination of vaccination against HPV and systematic screening. However, although its incidence has declined over the past decade, it remains the second leading cause of cancer deaths in women (Siegel et al., 2022).

CC can be managed with effective surgical techniques in the early stages, while in the advanced stages with more extensive disease or metastatic lesions, first-line treatment options include radiotherapy with concomitant chemotherapy with cisplatin, paclitaxel, and carboplatin and the addition of the angiogenesis inhibitor, bevacizumab, to platinum-based chemotherapy. In 2018, pembrolizumab, an immune checkpoint inhibitor, received FDA approval for the treatment of advanced recurrent CC following progression during or after standard chemotherapy despite severe inflammatory reactions (Wu et al., 2019; Walsh and Tan, 2021). Thus, when first-line treatments fail, second-line options are limited, confirming that the unmet medical need in CC is the identification of novel and effective therapeutic options.

In the era of precision medicine, some unique biological characteristics of a tumor, such as the impairment of specific cellular mechanisms, can suggest alternative therapeutic strategies.

Cervical carcinoma is among the five mutant homologous recombination (HR)-associated cancer types together with bladder cancer, endometrial cancer, head and neck cancer, and neuroendocrine tumor (Guo et al., 2020). More defects in homologous recombination deficiency (HRD) correlate with a higher prevalence of aggressive molecular features, and in addition, 25.2% of all cervical cancer tumors express the PTEN mutation (Mysona et al., 2021).

In cancer types characterized by DNA instability and deficient DNA repair system, such as ovarian and cervical cancers, trabectedin (Yondelis, ET-743), which directly interferes with DNA repair pathways and generates double-strand breaks (DSB), could be a promising therapeutic option. Regarding gynecological cancer, the European Medicines Agency approved trabectedin for the treatment of platinum-sensitive recurrent ovarian cancer in combination with pegylated liposomal doxorubicin in 2009.

Trabectedin is an inhibitor of oncogenic transcription. Its mechanism of action initiates after binding to certain areas on the minor groove of DNA. This binding triggers a cascade of effects affecting several transcription factors, DNA binding proteins, and DNA repair pathways, resulting in transcription inhibition, perturbation of the cell cycle, and apoptosis of the tumor cell. In addition, it shows immune-modulating activity through the inhibition of certain populations of myeloid tumor-associated cells. It has been shown that combined treatments of trabectedin and checkpoint inhibitors, such as pembrolizumab, produce an additive/synergistic antitumor effect in ovarian cancer models (Guo et al., 2015).

Recently, growing attention has been given to the effects of chronic stress on female reproductive physiology, which can accelerate tumor progression and are often related to a poor prognosis and higher mortality (Thaker et al., 2013; D’Incalci et al., 2014; Demetri et al., 2017). This is especially important in CC patients because they have a greater quality of life disruption than patients with other cancer types (Shylasree et al., 2021). Stress stimuli induce the activation of the sympathetic nervous system with a consequent increase of catecholamine release (epinephrine and norepinephrine) in the bloodstream and tissues, which bind the adrenergic receptors (ARs) (Thaker et al., 2006; Thaker et al., 2013). Although there is still limited evidence, β-adrenergic signaling also plays a key role in cervical cancer development and progression. β-Adrenergic receptors (β-ARs) are expressed in a broad spectrum of cancer types and are responsible for numerous stress-related responses in cancer cells and in the tumor microenvironment and for that, stress-activated mechanisms driving cancer could be abrogated by the β-adrenergic antagonists, such as propranolol (Thaker et al., 2006).

Thus, we investigated in both CC and ovarian cancer cell lines and in CC patient-derived organoids the antitumoral efficacy of trabectedin and evaluated if the repositioning of propranolol for the treatment of both CC and OC could potentiate trabectedin effectiveness, as it did for doxorubicin and docetaxel in *in vitro* models of soft tissue sarcoma (Porcelli et al., 2020).

Additionally, we tested the impact that these drugs have on the immune checkpoint PD-L1/PD1 expression level in tumor cells, with the future goal to investigate if immunotherapy with anti-PD1 could benefit from drug combinations with trabectedin and propranolol in CC. For this purpose, we utilized two CC cell lines which are the first from adenocarcinoma (HeLa cells with PIK3CA wt) and the latter from cervical squamous cell carcinoma (OV2008) with mutations in PIK3CA, which are reported to be associated with an immunosuppressive microenvironment (Walsh and Tan, 2021).

## 2 Materials and methods

### 2.1 Cell lines and culture conditions

Human cervical adenocarcinoma cell line HeLa (CCL-2), human ovarian adenocarcinoma cell line Caov-3 (HTB-75), and human ovarian adenocarcinoma ascites metastasis cell line SK-OV-3 (HTB-77) were purchased from ATCC (USA). Human cervical squamous cell carcinoma cell line OV2008 (ATCC) was generously provided by Prof. G.J. Peters (VUMC, Amsterdam, Netherlands). Caov-3 and OV2008 cell lines were cultured in Dulbecco’s modified Eagle’s medium (DMEM), while SK-OV-3 cells were cultured in McCoy’s 5a modified medium and HeLa in Eagle’s minimum essential medium (EMEM). All media were supplemented with fetal bovine serum (FBS) to a final concentration of 10%, L-glutamine 1% (v/v), and penicillin/streptomycin 1% (v/v). All cell lines were maintained at 37°C in a humid atmosphere of 95% air and 5% CO_2_. All reagents for cell culture were purchased from Euroclone (Italy) or ATCC (USA).

### 2.2 Patient-derived organoids

This study was performed in line with the principles of the Declaration of Helsinki. The surgical specimens were obtained from three patients with cervical carcinoma enrolled in an institutional protocol approved by the ethics committee of the University of Bari (Prot. 0070295 10/06/2020) and conducted in accordance with the international standards of good clinical practice. Informed consent was obtained for all three patients. Surgical specimens were minced in small pieces and digested in 1 mg/mL type IV collagenases for 1 h at 37°C. Then, the digestion was stopped by adding a double volume of 10% FBS culture medium, and the digested samples were passed through a 70 µm strainer and centrifuged. Afterward, the red blood cells were lysed by using ACK Lysing Buffer (GibcoTM, USA). The method followed and the growth medium prepared for the development of patient-derived organoids (PDOs) was as reported in Lõhmussaar et al. (2021). For the cytotoxicity study, the PDOs were counted and seeded in 96-well round-bottom low-attachment plates (Corning, NY, United States) and treated with 5 and 10 nM of trabectedin and 50 µM of propranolol. After 48 h, the morphological effects of the treatment on PDOs were assessed using a Celldiscoverer 7 microscope (Carl Zeiss Microscopy, Germany), and PDO viability was evaluated by Cell Titer-Glo^®^ 3D Cell Viability Assay (Promega, WI, United States), according to the manufacturer’s instructions.

### 2.3 Cell viability assay

The 3-[4,5-dimethylthiazol-2-yl]-2,5-diphenyltetrazoliumbromide (MTT) assay was performed in order to investigate the effect of trabectedin, propranolol, and their combined treatment on the viability of HeLa, OV2008, Caov-3, and SK-OV-3 cell lines in the presence and absence of 10 µM of norepinephrine (NE). Untreated cells were used as a negative control. Cells were seeded in 96-well plates at a density of 5,000 cells/well and incubated for 24 h in the culture medium. The cells were challenged with a scalar concentration of trabectedin (0.1–10 nM) or propranolol (10–100 µM) for 24 h. Then, 10 µL of 0.5% MTT was added to each well, and the plates were incubated until the medium was removed and replaced with DMSO (100 µL). The absorbance was measured using a microplate reader. The results are shown as a dose/effect plot representing the mean of three different experiments. The IC50 was defined as the drug concentration yielding a fraction of affected (no surviving) cells = 0.5, compared with untreated cells and was calculated utilizing CalcuSyn ver. 1.1.4 software (Biosoft, United Kingdom). For the combination study, the scalar concentration of trabectedin (0.1–10 nM for OV2008, Caov-3 and SK-OV-3 and 0.1–5 nM for HeLa cell line) was combined with a fixed dose of propranolol (50 µM in HeLa and Caov-3 and 100 µM in OV2008 and SK-OV-3 cell lines) in the absence or presence of NE (10 µM). The effect of the treatment on OV2008 spheroids was assessed by using the Celldiscoverer 7 microscope (Carl Zeiss Microscopy, Germany) after staining cells with MTT.

### 2.4 Cell cycle analysis

HeLa, OV2008, Caov-3, and SK-OV-3 cell lines were seeded in 60 mm dishes at a density of 300,000 cells/well and exposed to trabectedin (IC_50_) and/or propranolol (HeLa - Caov-3: 50 μM, OV2008 - SK-OV-3: 100 µM) alone or in combination in the absence or presence of 10 µM of NE. Afterward, the cells were harvested, washed twice in cold PBS, fixed in 70% ethanol, and stored at −20°C until analysis. The cell cycle modulation by drugs was evaluated by propidium iodide (PI) staining, as previously described (Danza et al., 2021). Then, flow cytometry (FCM) analysis was performed by using the Attune NxT Acoustic Focusing Cytometer (Thermo Fisher Scientific, MA, United States), and cell cycle phases were analyzed by the Attune NxT Analysis Software (Thermo Fisher Scientific, MA, United States).

### 2.5 Apoptosis assay

HeLa, OV2008, Caov-3, and SK-OV-3 cell lines were treated as described earlier. Then, the induction of apoptosis was determined by FCM using the FITC Annexin V Apoptosis Detection Kit II (BD Pharmingen, United States) according to the instructions provided by the manufacturer. The analysis was performed using the AttuneTM NxT Acoustic Focusing Cytometer (Thermo Fisher Scientific, MA, United States) and analyzed by using the AttuneTM NxT Analysis Software (Thermo Fisher Scientific, MA, United States). Data are presented as the percentage of Annexin V- and/or PI-positive cells.

### 2.6 γH2AX quantification

The phosphorylation of H2AX was evaluated by FCM analysis. Treated cells were processed and fixed as described earlier for cell cycle analysis, and the staining of the cells was performed as previously described (Iacobazzi et al., 2022). The antibodies utilized were as follows: IgG1 isotype control, the phospho-histone H2AX (Ser-139) antibody (Millipore, United States), and the goat anti-mouse IgG (H&L) fluorescein-conjugated affinity purified secondary antibody (BD Pharmingen, San Diego, CA, United States). The analysis was performed using the AttuneTM NxT Acoustic Focusing Cytometer (Thermo Fisher Scientific, MA, United States), and γH2AX quantification was obtained by the AttuneTM Analysis Software (Thermo Fisher Scientific, MA, United States).

### 2.7 Mitochondrial membrane potential evaluation by JC-1 staining

HeLa, OV2008, and Caov-3 cell lines were seeded as described earlier. Mitochondrial transmembrane potential after treatments was detected by staining cells with 5,5′,6,6′-tetrachloro-1,1′,3,3′ tetraethyl benzimidazolylcarbocyanine iodide/chloride (JC-1, Molecular Probes, Life Technologies, United States) cationic dye according to the manufacturer’s instructions. The analysis was performed using the AttuneTM NxT Acoustic Focusing Cytometer (Thermo Fisher Scientific, MA, United States), and the data were analyzed by the AttuneTM Analysis Software (Thermo Fisher Scientific, MA, United States).

### 2.8 Immunocytochemistry and immunofluorescence

Caov-3 and HeLa cells were seeded into glass Lab-Tek Chamber Slides (8 wells; 0.8 cm^2^/well) at a density of 25 × 10^3^/well and allowed to attach for 24 h at 37°C. After treatment with trabectedin and propranolol alone and in combination in the presence of 10 µM of NE, the cells were washed twice with PBS and fixed with 3.7% formaldehyde in PBS for 15 min at room temperature. For β-AR expression determination, the cells were incubated with diluted primary antibody anti-β1 adrenergic receptor (Abcam), anti-β2 adrenergic receptor (Abcam), and anti-β3 adrenergic receptor (Abcam) in 1% BSA in PBS for 1 h at RT. After three washing steps with PBS, the cells were incubated with EnVision + System-HRP labeled polymer secondary antibody (Dako, Denmark). Color development was obtained with AEC solution (Dako, Denmark), whereas the nuclei were counterstained with hematoxylin. Finally, the slides were sealed with the Kaiser mounting medium for optical observation and examined using a Leica DMi8 microscope. For RAD-51, COX-2, and p-p53 immunostaining, after fixation, the cells were permeabilized with Triton X-100 [0.1% (w/v)] in PBS for 5 min at room temperature. Non-specific binding sites were blocked for 30 min at room temperature with 5% bovine serum albumin (BSA) in PBS, and then, the cells were incubated with a mouse anti-RAD-51 or a mouse anti-COX-2 (clone CX229) monoclonal antibody and rabbit anti-p-p53 (Ser15) monoclonal antibody in PBS containing 4% BSA for 60 min at room temperature. p-p53 and COX-2 immunostaining was followed by incubation with Alexa fluor 488 goat anti-mouse antibody (Invitrogen) for RAD51 and Alexa fluor 488 goat anti-rabbit antibody (Invitrogen) and Alexa fluor 568 goat anti-mouse antibody (Invitrogen) for p-p53 and COX-2, respectively, for 60 min at room temperature. After three washing steps with PBS, the slides were mounted on Vectashield with DAPI (Vector Laboratories) and examined using a Leica DMi8 microscope.

### 2.9 Cell target analysis by Western blotting

After 24 h of treatment, Caov-3, HeLa, and OV2008 cells were harvested and lysed on ice in cell lysis buffer (Cell Signaling Technology, MA, United States) and analyzed as described by Porcelli et al. (2021). In brief, 50 µg of proteins were electrophoretically separated on Mini-Protean TGX Precast Gels (Bio-Rad Laboratories, United States) by SDS-PAGE. The proteins were then transferred to PVDF membranes using the Trans-Blot Turbo Mini PVDF Transfer Packs (Bio-Rad Laboratories, United States). The membranes were incubated with primary antibodies (COX-2, (Cayman Chemical, United States), p-p44/42, p44/42, AKT, p-AKT, and p-p53(Ser15) (Cell Signaling, United States), and β-actin (Sigma, United States)) at 4°C overnight and HRP-conjugated secondary antibodies; EC Clarity Western ECL Substrate was used for antibody detection (Bio-Rad Laboratories, United States). Images were captured using ChemiDoc (Bio-Rad Laboratories, United States) and analyzed by ImageLab software (ver. 5.2.1).

### 2.10 Gene expression analysis by quantitative real-time PCR

In order to evaluate the effect of the drugs on COX-2 mRNA expression, the cells were treated with trabectedin, propranolol, and their combined treatment for 24 h in the presence of 10 µM of NE. Then, the treated cells were harvested, total RNA was extracted using TRIzol reagent (Thermo Fisher Scientific, MA, United States), and the concentrations were quantified with the ND8000 Spectrophotometer (NanoDrop Technologies). Then, 500 ng of total RNA was reverse-transcribed using the High-Capacity cDNA Reverse Transcription Kit (Applied Biosystems™), and quantitative real-time PCR (qPCR) was performed on the StepOnePlus™ Real-Time PCR System (Applied Biosystems™) by using COX-2 and GAPDH-specific primers (Roelofs et al., 2014) and PowerUp™ SYBR™ Green Master Mix mRNA quantitative real-time polymerase chain reaction Kit (Applied Biosystems™) according to the manufacturer’s instructions. Gene expression levels were quantified by the comparative ΔΔCt method after normalization for the endogenous reference (GAPDH). All the PCR reactions were performed in duplicate three times.

### 2.11 FCM for PD1 and PD-L1 expression analysis in OC and CC cell lines

PD1 and PD-L1 expression analysis was performed by FCM in OC and CC cell lines at the basal level and after 24 h treatment with trabectedin and propranolol alone and in combination under stress stimuli by 10 µM of NE. Cells were seeded in 60 mm dishes at a density of 500,000/well and treated after 24 h. Afterward, the cells were harvested, washed twice, resuspended in ice-cold PBS without Ca^2+^ and Mg^2+^, and incubated to labeling with anti-human SuperBright 702-PD1 and anti-human PE-Cyanine 5-PD-L1 antibodies for 30 min at 2°C–8°C in the dark. After staining, the cells were washed with PBS without Ca^2+^ and Mg^2+^ and analyzed by using the Attune NxT Acoustic Focusing Cytometer (Thermo Fisher Scientific, Waltham, MA, United States) equipped with four lasers (405 nm (violet), 488 nm (blue), 561 nm (yellow), and 637 nm (red)) for sample reading. The data were analyzed using the Attune NxT Analysis Software (Thermo Fisher Scientific, Waltham, MA, United States).

### 2.12 Statistical analysis

The statistical significance was calculated using two-tailed t-tests, while the analysis of variance was performed by using a two-way ANOVA, followed by Bonferroni *post hoc* tests (GraphPad Prism version. 5.0). Results were reported as the mean ± SD of three independent experiments (*p < 0.05, **p < 0.01, and ***p < 0.001).

## 3 Results

### 3.1 Antitumor activity of trabectedin in CC cell lines

**FIGURE 1:**
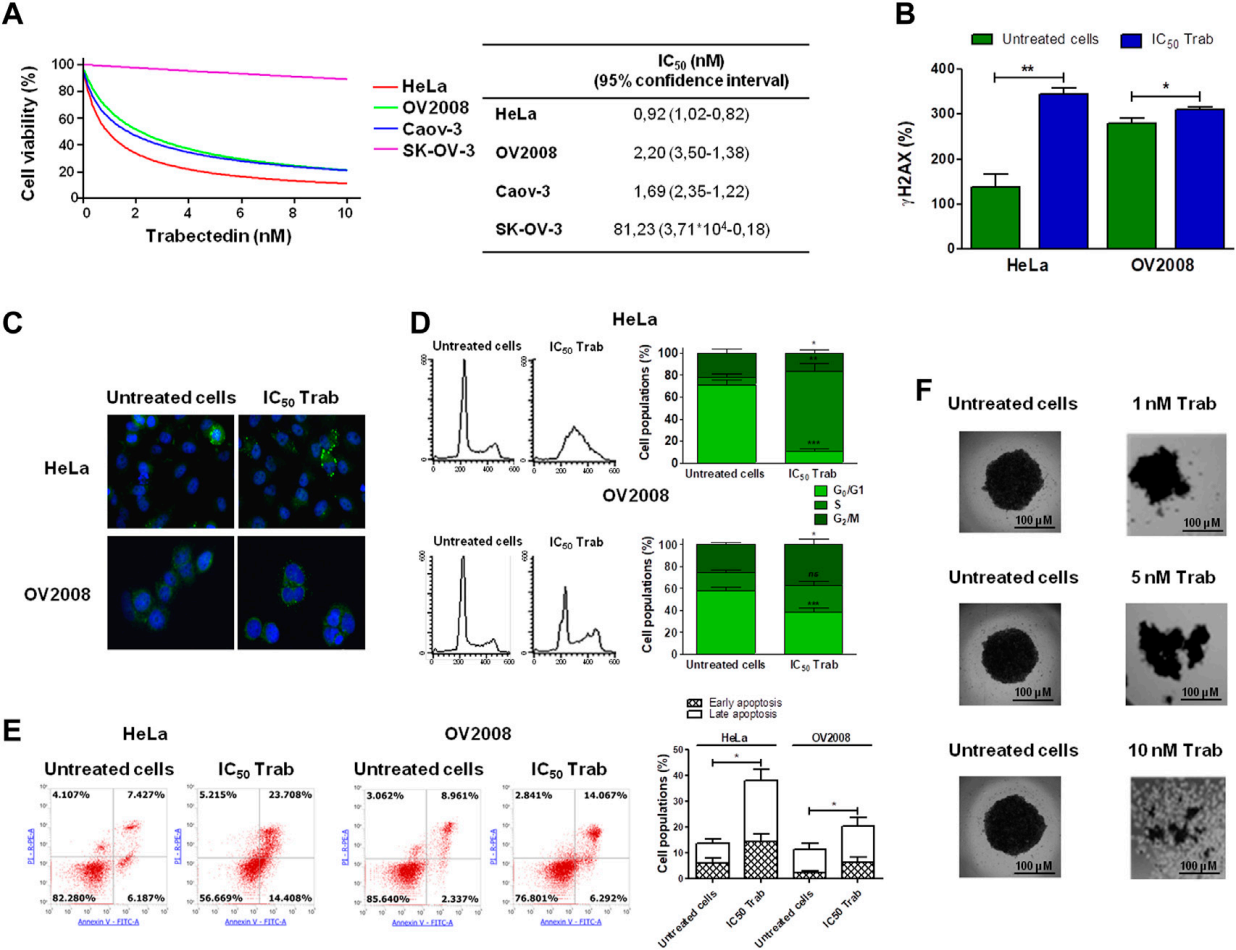
Evaluation of trabectedin cytotoxic effects. **(A)** Ovarian and cervical cancer cell lines were incubated with increasing concentrations of trabectedin, and cell viability was evaluated after 24 h. The results are reported in a dose/effect plot. The IC_50_ values were calculated utilizing CalcuSyn ver. 1.1.4 software; **(B)** histogram plots showing the phosphorylation of H2AX (γH2AX+ cells %), analyzed by FCM and reported as mean ± SD; **(C)** representative IF images showing cytosolic RAD51 foci formation; **(D)** representative plots showing the perturbation of cell cycle progression, analyzed by FCM, and results are reported as histogram plots of mean ± SD; **(E)** dot plots showing Annexin V/PI-positive cells, analyzed by FCM, and histogram plots reporting early and late apoptosis quantification (mean ± SD); and **(F)** representative images showing trabectedin cytotoxicity in OV2008 spheroids. *p < 0.05, **p < 0.01, and ***p < 0.001.

As an innovative pharmacological approach in the treatment of CC, we investigated the efficacy of trabectedin in two CC models, one primitive (HeLa—cervical adenocarcinoma) and the other metastatic (OV2008—cervical squamous cell carcinoma), and in two OC models, Caov-3 (primary tumor) and SK-OV-3 (metastatic one), that were used as reference models to assess drug cytotoxicity. All cell lines were incubated with increasing concentrations of trabectedin. Such treatment reduced the number of viable cells in a dose-dependent manner, with IC_50_s ranging from 0.92 to 81.23 nM. Both CC cell lines were very sensitive, and so was the primitive OC Caov-3 cell line, while the metastatic OC cell line was not very responsive to trabectedin. Notably, the cytotoxicity study evidenced that the metastatic cell lines (OV2008 and SK-OV-3) were less susceptible to the activity of trabectedin compared to the primitive ones. The dose-response plots and IC_50_ values are summarized in Figure 1A. To confirm that trabectedin utilized its well-known cytotoxic mechanism of action in CC models also, the ability of the drug to cause DNA DSBs, to affect cell cycle progression, and to induce apoptosis was investigated. Hence, we determined as surrogate marker of DSB, induced by Trabectedin, the phosphorylation of H2AX (ɣ-H2AX), and investigated whether this event led to the formation of RAD51 foci, which is regarded as a functional assay for testing the activation of homologous recombination (HR) in ovarian cancer (Gerić et al., 2014; van Wijk et al., 2022; Blanc-Durand et al., 2023). Trabectedin at its IC_50_ induced an increase of the phosphorylation of H2AX of 2.5- and 1.1-fold versus untreated cells in HeLa and OV2008 cells, respectively (Figure 1B). However, as shown in Figure 1C, trabectedin did not allow the formation of RAD51 foci in the nucleus of both HeLa cells and OV2008 cells, suggesting that, upon DSB, the cells failed to trigger the DNA damage response (DDR) through the HR repair pathway. Notably, we observed the formation of cytosolic RAD51 foci preferentially in the HeLa cell line than in the OV2008 cell line. According to DNA damage caused by trabectedin and stalled replication fork induction, the drug yielded an evident block of cells in the S-phase, as shown in Figure 1D, in which the cell cycles of CC cells are reported. Following S-phase arrest, the cells underwent apoptosis with an increase of 2.6- and 1.8-fold over untreated cells in HeLa and OV2008 cell lines, respectively (Figure 1E). Finally, we evaluated trabectedin effectiveness in an *in vitro* 3D spheroid model, which allows the cells to develop a more elaborate extracellular matrix and better intercellular communication. The spheroids were obtained by incubating OV2008 cells for 5 days in low-attachment plates, and after incubation with trabectedin for 48 h, in a range between 1 and 10 nM, a dose-dependent reduction of viable cell (purple cells) was found (Figure 1F).

### 3.2 Validation of trabectedin effectiveness in *ex vivo* models of CC

**FIGURE 2:**
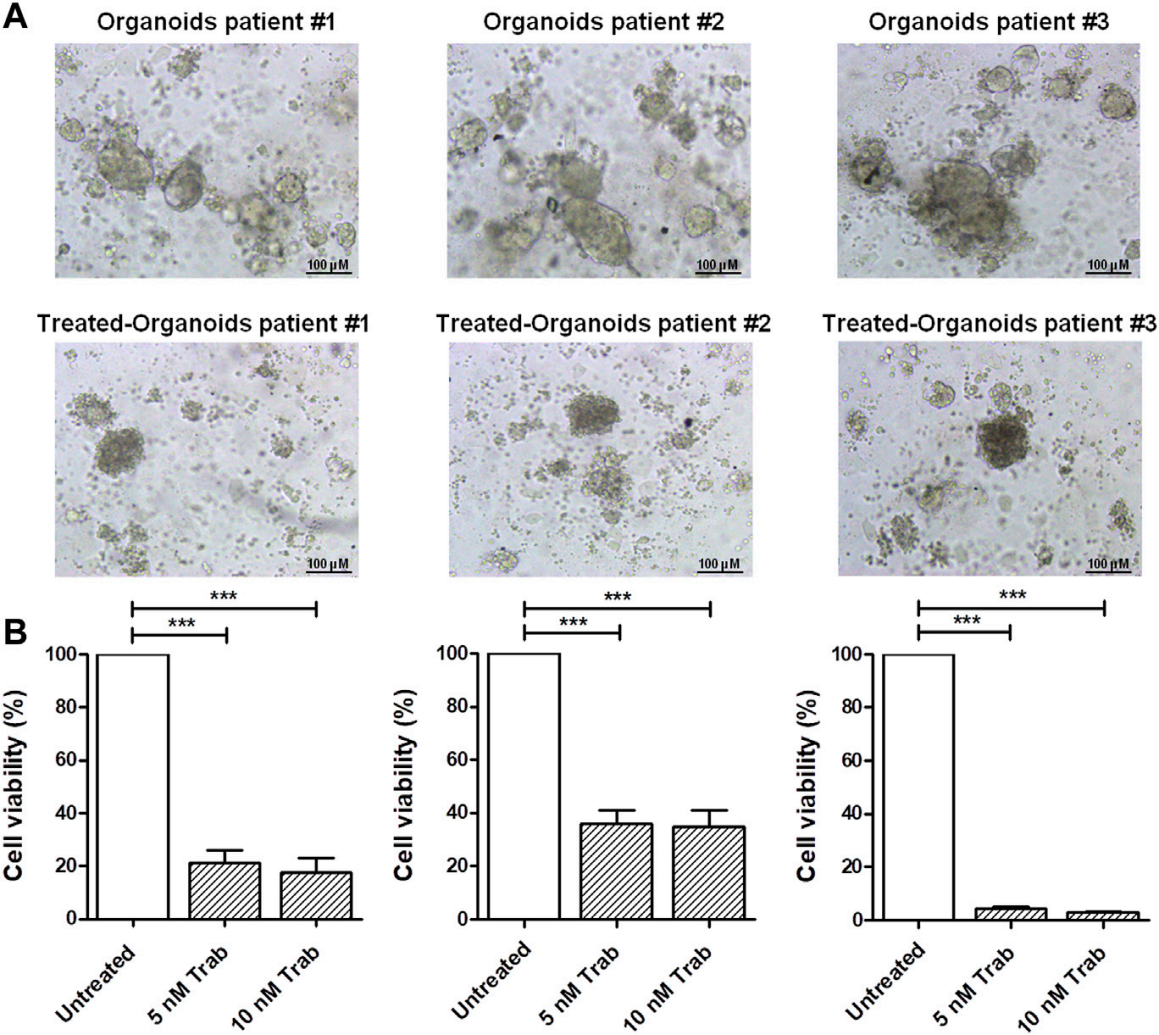
Viability assay in CC *ex vivo* models validates the effectiveness of trabectedin treatment. **(A)** Representative images of pz-1-, pz-2-, and pz-3-PDOs untreated and treated with 5 and 10 nM of trabectedin (48 h); scale bar 100 µm. **(B)** Histogram plots showing the results of cell viability (%), reported as mean ± SD of three independent experiments; ***p < 0.001.

In order to provide translational evidence on the use of trabectedin in CC, we used *ex vivo* models. For this purpose, we established PDOs from three CC patients’ tumor tissues collected and immediately processed at the time of surgical resection. As shown in Figure 2A, the incubation of PDOs with 5 and 10 nM of trabectedin for 48 h affected the compactness and strongly reduced the viability of the PDOs by at least 65% compared to the untreated ones, in a slightly concentration-dependent manner (Figure 2B), confirming the promising use of trabectedin in CC treatment**.**

### 3.3 The targeting of β-ARs enhances trabectedin efficacy in CC and OC models

**FIGURE 3:**
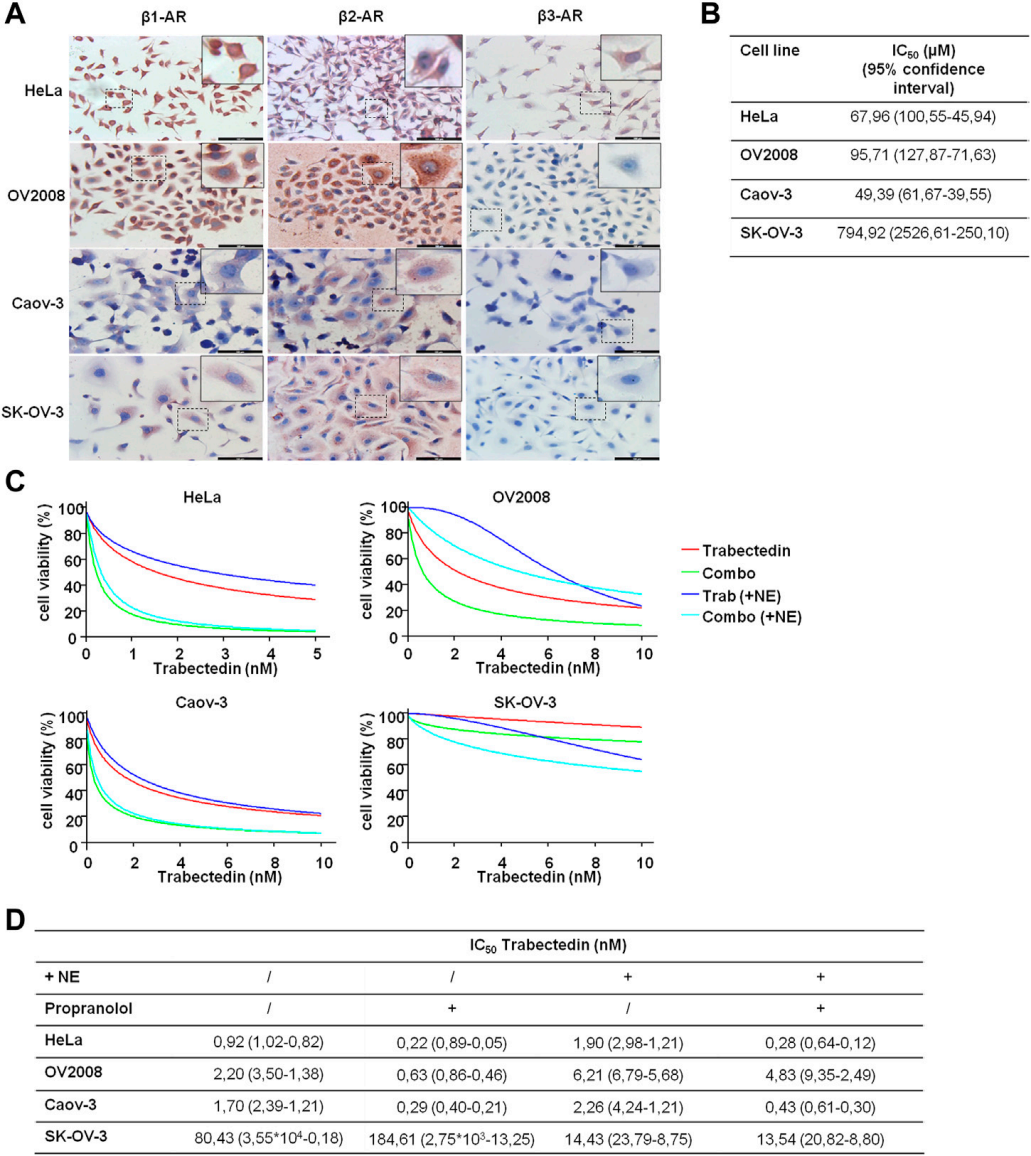
β-AR isoform expression in OC and CC models supports the use of propranolol to increase trabectedin effectiveness even in NE-mediated stress conditions. **(A)** Representative images of basal level expression of β-ARs, evaluated by ICC, in OC and CC cell lines; scale bar 100 µm. **(B)** IC_50_ values obtained after exposure of OC and CC cell lines to increasing concentrations of propranolol for 24 h in the presence of 10 µM of NE and calculated by using CalcuSyn ver. 1.1.4. **(C)** Dose–effect plots of the results of viability assay conducted in OC and CC cell lines after 24 h exposure to IC_50_ trabectedin alone or in combination with propranolol in the absence or presence of 10 µM of NE. **(D)** Trabectedin IC_50_ values (95% confidence interval) in all conditions tested, calculated by using CalcuSyn ver. 1.1.4.

Since we hypothesized that propranolol, by targeting β-ARs, could increase the efficacy of trabectedin, we preliminary evaluated β-ARs expression in the two models of CC and in the two models of OC. The three isoforms, β-AR types 1, 2, and 3, were evaluated by ICC, and the images are shown in Figure 3A. The OV2008 cell line and both OC cell lines showed a moderate or weak staining for β1-AR, respectively, a stronger staining for β2-AR, and an undetectable staining for β3-AR. Instead, HeLa cells displayed a strong expression of β1-ARs and moderate and weak expressions for β2-AR and β3-AR, respectively. Overall, in all cell lines, the ICC analysis revealed that β-ARs were mainly localized at the cytoplasmic level.

To determine the impact of the β-AR signaling-dependent tumorigenic effect on trabectedin effectiveness, we used the non-selective β-blocker propranolol and carried out all experiments in the presence of NE to simulate the physiological condition in which chronic stress induced the release of this catecholamine. NE was utilized at 10 μM, as suggested by Barbieri et al. (2015). We evaluated propranolol activity as inhibitor of cell viability by utilizing the β-blocker at increasing concentration; the data are reported as IC_50_s (Figure 3B). In the evaluation of the propranolol-dependent modulation of trabectedin efficacy, the β-blocker was used at 50 µM in the HeLa and Caov-3 cell lines and at 100 µM in the metastatic cell lines, OV2008 and SK-OV-3.

In Figures 3C, D, the modulation of trabectedin efficacy by propranolol in the absence or presence of NE is shown in order to clarify whether the stress condition reduced the efficacy of trabectedin and whether it is recovered with the addition of propranolol. First, the stress induced by NE reduced cell response to trabectedin of about 1.3–3-fold in all cell lines (in terms of IC_50_ increase), except in SK-OV-3 cells, in which a strong increase of 5.6-fold trabectedin effectiveness was found in terms of trabectedin IC_50_ reduction. The addition of propranolol strongly increased trabectedin effectiveness, as evident in Figure 3C (light blue vs. blue), with a reduction of IC_50_ values of 4.18-, 3.49-, and 5.83-fold in HeLa, OV2008, and Caov-3 cell lines, while in SK-OV-3 cells, an increase of 2.27-fold was found. Collectively, in the presence of NE, the addition of the β-blocker increased the response to trabectedin in all cell lines, as shown by the decrease of trabectedin IC_50s_ values (Figure 3D).

Since OV2008 cells showed greater sensitivity to propranolol than SK-OV-3, we also tested the combination by using the 50 µM β-blocker (data not shown). No appreciable differences emerged with respect to 100 µM of propranolol and, therefore, in Figure 3C, we reported the plots related to the lowest concentration which was also used in subsequent experiments.

### 3.4 Validation of trabectedin plus propranolol effectiveness in CC PDOs

**FIGURE 4:**
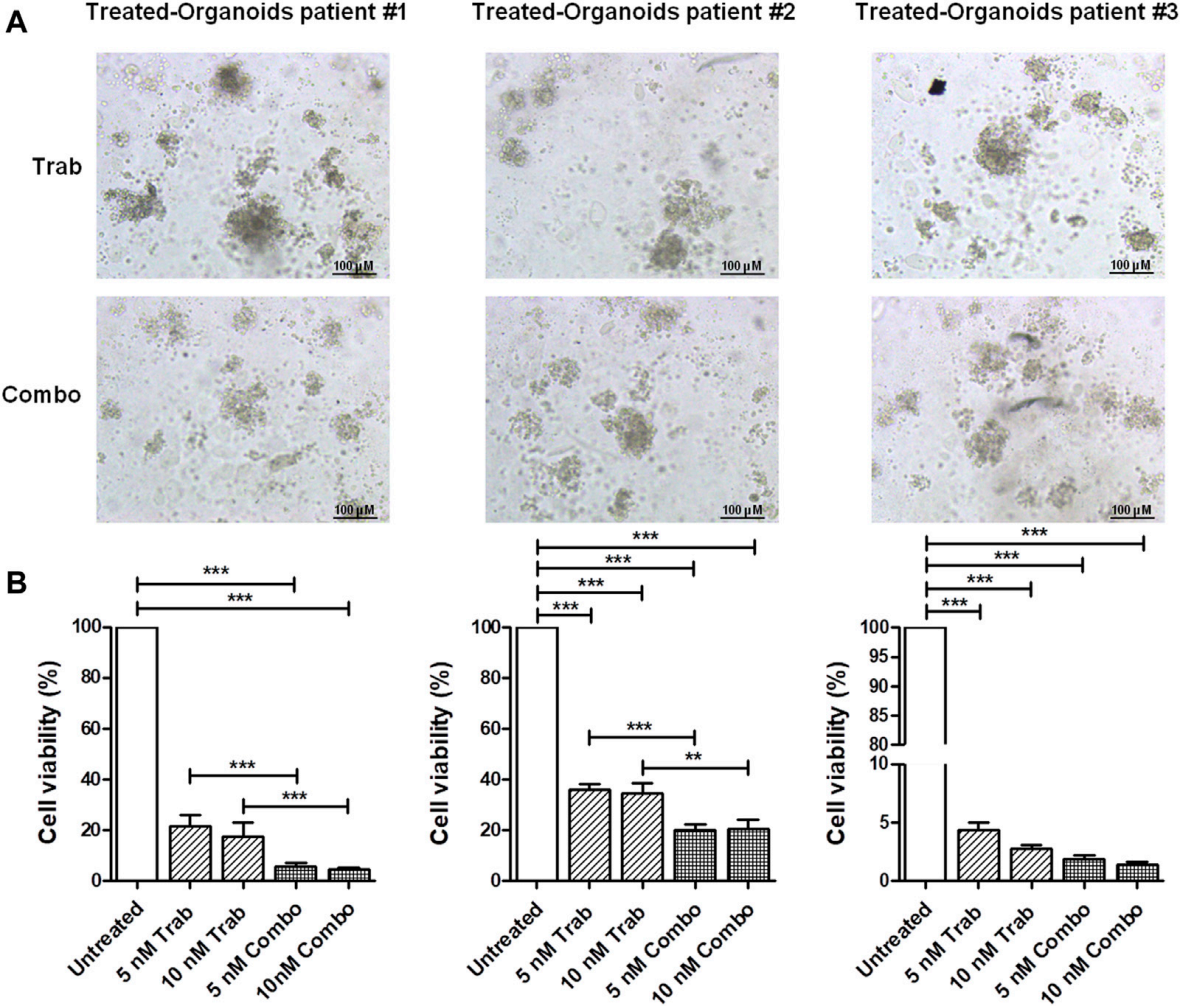
Viability assay in CC *ex vivo* model corroborates the efficacy of combination treatment. **(A)** Representative images of pz-1-, pz-2-, and pz-3-PDOs treated with 10 nM of trabectedin alone or combined with propranolol for 48 h; scale bar 100 µm. **(B)** Histogram plots of cell viability (%) reported as the mean ± SD of three independent experiments; **p < 0.01; ***p < 0.001.

We validated the increase in the efficacy of the combination of trabectedin and propranolol in the three *ex vivo* models, observing a further reduction in the viability of CC PDOs (Figure 4) by the addition of the β-blocker. The images of PDOs reported in Figure 4A show their reduction in size when incubated with the two drugs in combination for 48 h, which seemed to be slightly trabectedin dose dependent. PDO viability values are reported as histogram plots in Figure 4B The addition of propranolol to 5 nM of trabectedin induced a further reduction of PDO viability from 21.3% to 5.4%, 36.0% to 20.4%, and 4.4% to 2.9% in pz1-PDOs, pz2-PDOs, and pz3-PDOs, respectively, with a difference statistically significant in PDOs-pz1 and PDOs-pz2 (Figure 4B).

### 3.5 Trabectedin plus propranolol reduces CC viability by hindering DNA repair

Since the combination of trabectedin and propranolol revealed to be a promising pharmacological strategy in both CC and OC models, we investigated how the addition of the β-blocker modified trabectedin activity in terms of cell cycle progression perturbation and RAD51 foci formation in the presence of NE.

**FIGURE 5:**
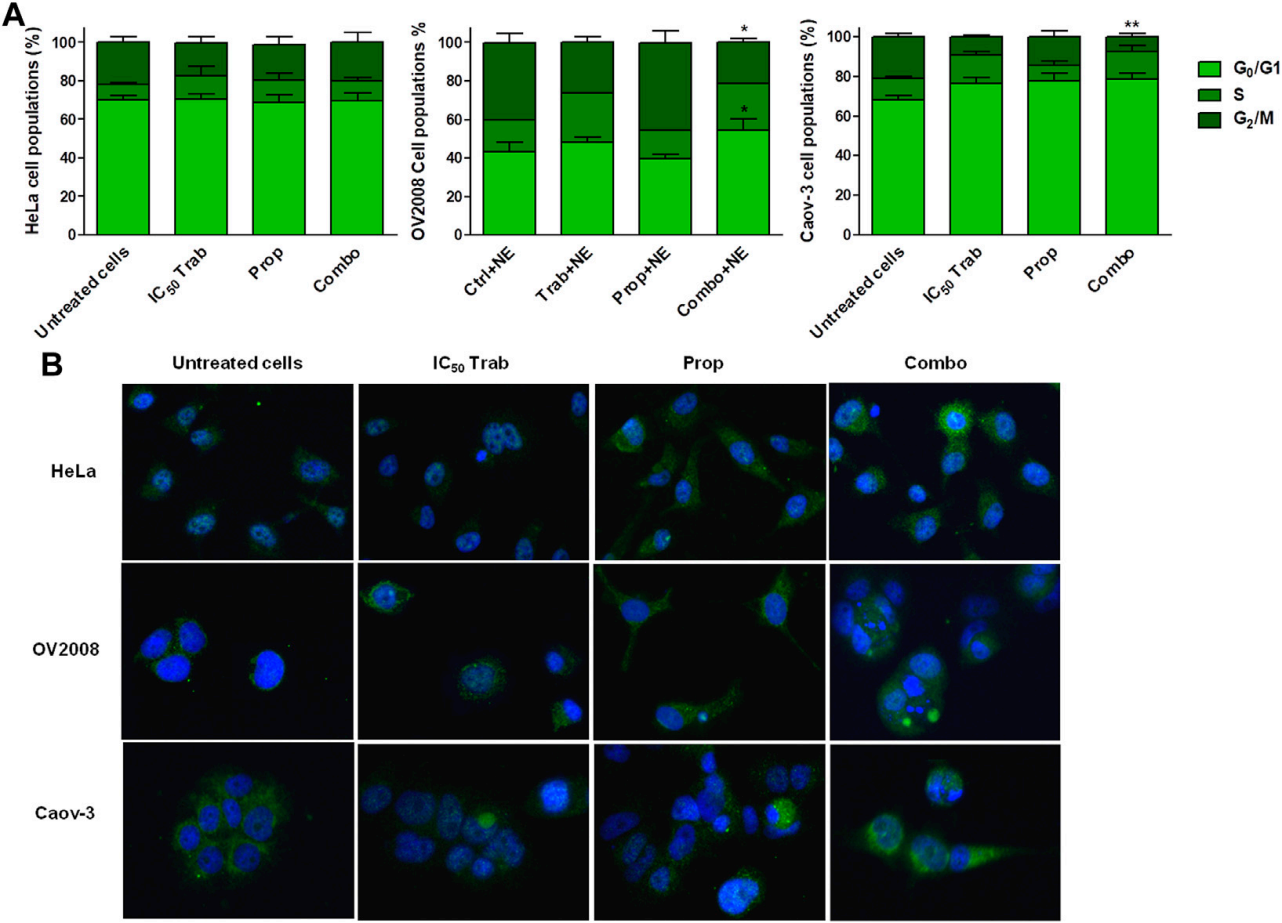
Stress stimuli and the addition of propranolol modify Ttabectedin effects in terms of cell cycle modulation, induction of DNA damage, and RAD51 foci formation. **(A)** Histogram plots showing cell cycle modulation in HeLa, OV2008, and Caov-3 cell lines in all treatment conditions in the presence of 10 µM of NE; the results are reported as mean ± SD of three independent experiments. *p < 0.05 (Combo+NE-G0/G1 vs. Prop+NE-G0/G1 and Combo+NE-G2/M vs. Prop+NE-G2/M in OV2008); **p < 0.01 (Combo+NE-G2/M vs. Untreated+NE-G2/M in Caov-3). **(B)** Representative IF images showing no RAD51 foci formation in the nuclei (blue, DAPI: nuclei; green: RAD51).

The stimulation with NE reduced the trabectedin-dependent S-phase arrest in CC and OC cells, and the addition of propranolol did not consistently modify the cell cycle progression in HeLa cells; instead, it slightly increased the G0/G1 phase in OV2008 and reduced the G2/M phase in both OV2008 and Caov-3 cell lines (Figure 5A). The analysis of RAD51 foci formation showed that trabectedin caused the mislocalization of RAD51 in the cytosol, and the propranolol seemed to further reduce its localization in the nucleus, either when given alone or in combination with trabectedin (Figure 5B) in all cell lines. Collectively, such results suggested that under stimulation with NE, the addition of propranolol further abrogated the activation of HR and promoted cell cycle transition by removing the S-phase checkpoint.

### 3.6 Molecular mechanisms underlying tumor inhibition and apoptosis induction by treatments

**FIGURE 6:**
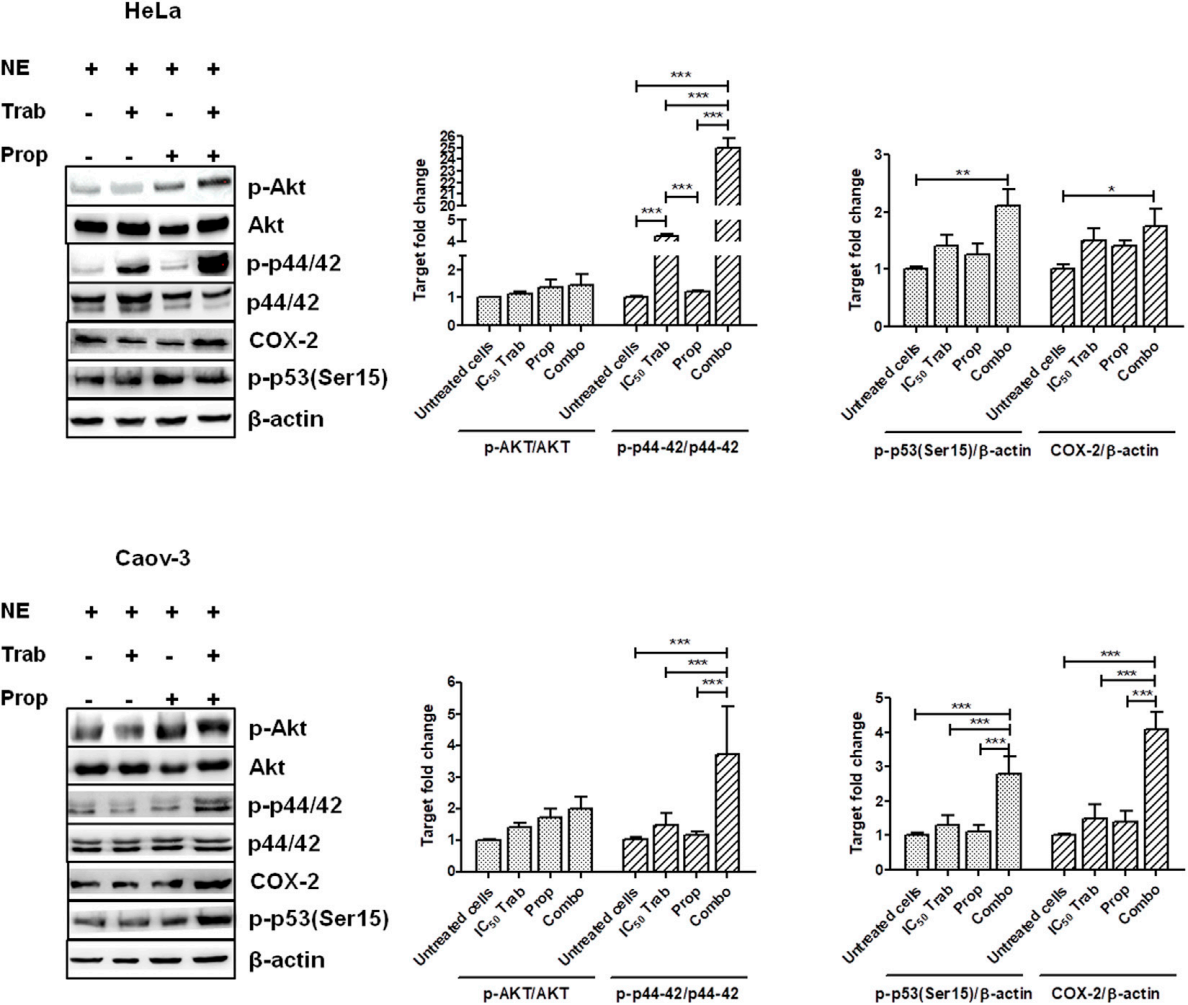
Treatment(s) affect cell targets related to apoptosis, proliferation, and survival signaling. Representative immunoblots showing the protein expression of targets upon treatment(s) and protein expression quantification reported in histogram plots. *p < 0.05, **p < 0.01, and ***p < 0.001.

**FIGURE 7:**
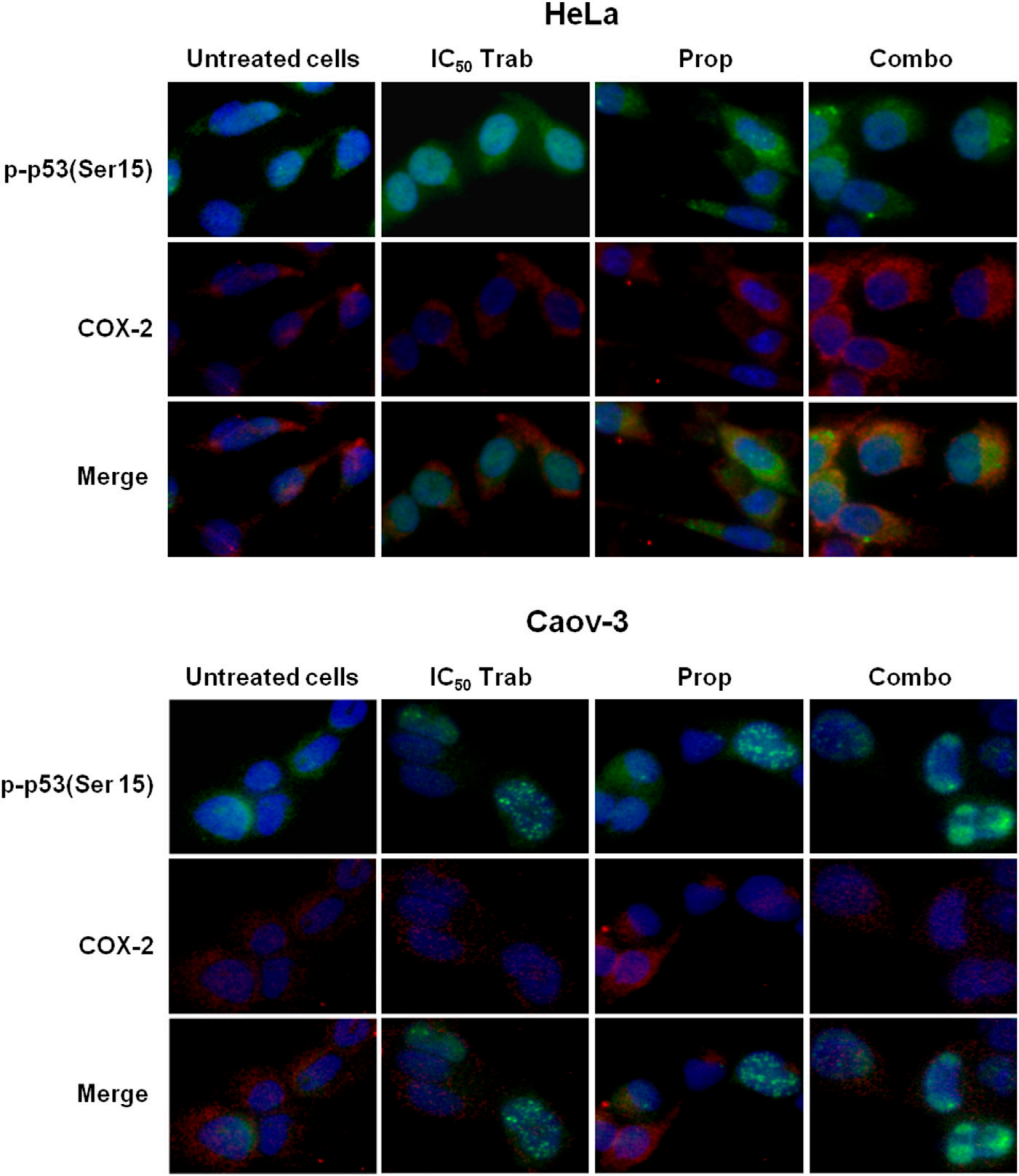
Propranolol modifies the trabectedin-dependent activation and localization of p-53. Representative IF images showing p-p53(Ser15) and COX-2 localization in HeLa and Caov-3 cell lines after 24-h exposure to IC_50_ trabectedin and propranolol alone or in combination under stress stimuli by 10 µM NE.

**FIGURE 8:**
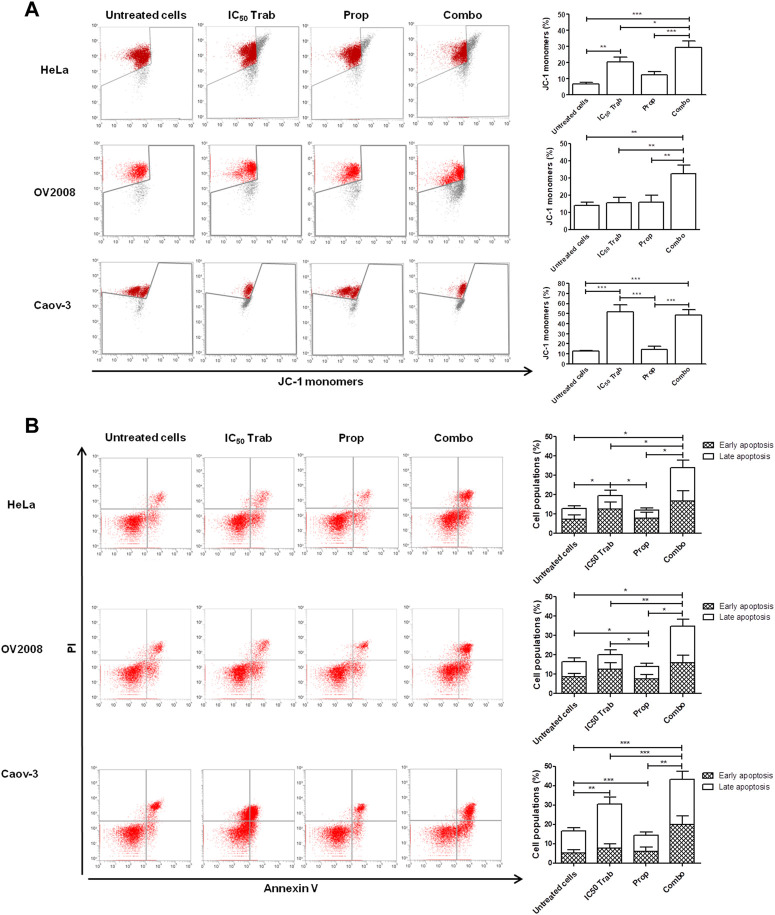
Addition of propranolol to trabectedin increases apoptosis and induces mitochondrial membrane depolarization in OC and CC cell lines. **(A)** Dot plots of JC-1 analysis showing the increase of JC-1 monomers after 24 h exposure to IC_50_ trabectedin or/and propranolol in HeLa, OV2008, and Caov-3 cell lines. The results are reported as mean ± SD of three independent experiments in histogram plots. **(B)** Dot plots reporting Annexin V/PI cells fraction evaluated in HeLa, OV2008, and Caov-3 cell lines after 24 h of all treatment conditions under 10 µM of NE stress stimuli, whose results are reported in histogram plots as mean ± SD of three independent experiments. *p < 0.05, **p < 0.01, and ***p < 0.001.

Because MAPK activation concomitant with an increase in the expression of COX-2 and p53 phosphorylation has been regarded as a mechanism of apoptosis induction in ovarian cancer cell lines by antitumor treatments (Lin et al., 2011; Lin et al., 2013), we assessed the phosphorylation of p44/42 and the expression of COX-2 after single-agent and combined treatment. We found that in HeLa and OV2008 cell lines, both trabectedin and, to a higher extent, the combined treatment induced the increase of p-p44/42 protein expression (Figure 6; Supplementary Figure S1A), while in Caov-3 cells, the increase of p44/42 activation was induced only by the combination (Figure 6). Accordingly, it was the combination of propranolol and trabectedin to increase the mRNA (Supplementary Figure S1B) and COX-2 protein expression (Figure 6; Supplementary Figure S1A), which resulted a significantly upregulated respect to the level found in the untreated cells and the propranolol-treated cells in all cell lines. As it has been demonstrated that COX-2 interacts with p53 and interferes with p53-dependent apoptosis induction (Corcoran et al., 2005), we performed IF evaluations to detect the cellular localization of both p53 and COX-2 in single-drug and combination-treated cells. In HeLa cells, following trabectedin treatment, p-p53(Ser15) was increased at the nuclear level, while the treatment with propranolol and with the combined therapy caused the accumulation of p-53 in the cytoplasm, where it co-localized with COX-2. Unlike HeLa cells, in Caov-3 and in OV2008 cell lines, both single-treatment and, to higher extent, the combined tratment induced remarkable nuclear p-53 activation, while COX-2 retained a cytoplasmic localization both in untreated and drug(s)-treated cells (Figure 7 and Supplementary Figure S2). By evaluating the expression of p-p53(Ser15) by WB, we found that trabectedin and, to higher extent, the combined treatment induced the increase of protein expression (Figure 6; Supplementary Figure S1A). In order to understand if the cytosolic localization of COX-2 and p53 in HeLa cells was associated with a change in mitochondrial potential and activation of the mitochondrial-stress induced apoptotic pathway, we exposed the cells to the cationic dye JC-1 and visualized the mitochondrial membrane depolarization (Salido et al., 2007) as a reduction in the fluorescence signal of JC-1 dimers and an increase of JC-1 monomers through FCM analysis. In the HeLa cell line, we detected a strong increase in green fluorescent monomeric form after exposure to trabectedin and even more after the combined treatment, while in OV2008 cells, the combined treatment caused the increase of mitochondrial membrane depolarization with respect to the single-drug treatment. In the Caov-3 cells, the addition of propranolol did not modify the already high level of the trabectedin-induced mitochondrial membrane depolarization (Figure 8A). This evidence confirmed the mitochondria involvement in the trabectedin-dependent antitumor effects in HeLa, OV2008, and Caov-3 cell lines. By staining the cells with Annexin V, we determined apoptosis induction by the treatment(s). We found that the combined treatment increased the percentage of cells in early and late apoptosis with respect to trabectedin-treated cells of 12.47%–6.86% vs. 16.75%–17.09%, 7.82%–22.65% vs. 19.87%–23.35%, and 12.73%–7.36% vs. 15.8%–18.88% in HeLa, Caov-3, and OV2008 cell lines, respectively (Figure 8B). Additionally, we investigated the involvement of Akt, among possible survival signaling pathway affected by treatments. Notably, propranolol increased the phosphorylation of Akt, and this activation was stronger in the combined therapy, as shown in Figure 6 and Supplementary Figure S1A, in which are reported the immunoblots performed in HeLa and Caov-3 and OV2008 cell lines.

### 3.7 Trabectedin and propranolol affected the PD1 expression level in CC and OC models

The PD-L1 is an FDA-approved predictive biomarker in cervical cancer with positive tumors eligible for single-agent pembrolizumab after progression on first-line chemotherapy (Walsh and Tan, 2021), while PD-L1 showed to be a valid predictive biomarker in OC (Zhu et al., 2021).

In order to evaluate if immunotherapy with anti-PD1 could benefit from the combined regimen with trabectedin and propranolol, we evaluated the impact that both drugs have on the checkpoint expression (Thaker et al., 2013).

The FCM analysis showed that the basal PD-L1 levels were 26.23%, 80.85%, 98.94%, and 29.54% in HeLa, OV2008, Caov-3, and SK-OV-3, respectively (Supplementary Figure S3A). The analysis in CC cells showed that HeLa cells had a lower expression level than OV2008 cells, confirming that in cervical adenocarcinoma, PD-L1 is less expressed than in cervical squamous cell carcinoma (Walsh and Tan, 2021). We exposed all cell lines to trabectedin and/or propranolol for 24 h, and no modulation in the expression level of PD-L1 was found, which is in agreement with Guo et al. (2015), in which the authors showed that the modulation and, consequently, the effect of ICI was evident only in *in vivo* models.

The basal levels of PD1 were 15.64%, 21.97%, 37.87%, and 11.91% in HeLa, OV2008, Caov-3, and SK-OV-3, respectively (Supplementary Figure S3B). In OV2008 and in the two OC cell lines, both drugs reduced the expression of PD1, and when given together, the reduction was more consistent. Conversely, in HeLa cells, the addition of propranolol increased PD1 expression by 1.54-fold and that of trabectedin by 1.67-fold, while, given together, the drugs caused an increase of 3.38-fold. These results suggested that only a direct evaluation of the effect that the combination of trabectedin and propranolol could have on ICI response can clarify whether this therapy is also immunomodulatory.

## 4 Discussion

We hypothesized that CC could benefit from the treatment with trabectedin since it is regarded as a tumor displaying high DNA instability and DNA repair system deficiency. In agreement with this hypothesis, we observed that both CC cell lines were very sensitive to trabectedin. Instead, between the two OC cell lines tested, only the Caov-3 cell line displayed a comparable sensitivity to the CC cell lines. We demonstrated that trabectedin inhibited cell viability at nanomolar concentrations, and its mechanism of action was similar to that already reported in other pathologies, e.g. by arresting cells in S and S-G2 phases of cell cycle and by inducing apoptosis. Notably, upon treatment with trabectedin, the cells failed the formation of the nuclear RAD51 foci, despite the activation of DDR, demonstrated by the increase of the phosphorylation of the Ser-139 residue of the histone variant H2AX. Further evidence confirming the efficacy of trabectedin in CC was obtained by testing the antitumor potential of the drug in PDOs, in which the drug exerted antitumor effect comparable to that found in the *in vitro* tumor models. In recent years, scientific research has focused its efforts on better understanding of cellular and molecular mechanisms responsible for the stimulatory effects of catecholamine on tumor growth. Such molecules can regulate cancer biology and processes involved in the tumor progression thanks to the interaction and subsequent activation of β-ARs that are widely expressed on tumor cells (Cole and Sood, 2012; Thaker et al., 2013; Rains et al., 2017).

In this perspective, we evaluated, in both the CC and OC models, whether the stress condition to which women are subjected during the management of gynecological tumors may impact the response to trabectedin and if it is possible to optimize its use by adding a β-blocker.

We demonstrated that β-ARs were highly expressed in both ovarian and cervical cancer cell lines that we tested, providing the rationale for using the non-selective β-ARs antagonist propranolol to counteract the NE-dependent pro-survival stimuli and to enhance the response to trabectedin. The stimulation with NE reduced the effectiveness of trabectedin in the whole range of concentrations utilized, especially in CC cell models. Instead, the combined treatment with propranolol resulted in greater antitumor efficacy in both OC and CC cell lines. This behavior was also observed in CC PDOs, in which the addition of propranolol further increased the effectiveness of the chemotherapeutic agent. Recent studies have suggested that stress mediated by NE can induce DNA damage by impairing DNA repair capacity and promoting cellular transformation and tumor cell proliferation (Lamboy-Caraballo et al., 2020). We found that even under stress stimuli, the drugs caused mislocalization of RAD51, resulting in unresolved genomic lesions and high levels of replication stress, which culminated with apoptosis induction. Notably, in agreement with Plo et al. (2008), we found that the cytoplasmatic retention of RAD51 correlated with activated Akt, allowing hypothesizing this as a possible mechanism leading to the repression of HR in treated cells. According to apoptosis induction, we found the increase of phosphorylation of p53 at Ser15, an event occurring rapidly in response to DNA double-strand breaks and carried out by ATM (Liu and Kulesz-Martin, 2001; Lavin and Gueven, 2006), and that highlighted the DNA damage caused by trabectedin and by the combination treatment with propranolol. Interestingly, the phosphorylation of p53 was accompanied by the increase of the expression of inducible COX-2 and p44/42 phosphorylation in both OC and CC tumor models, which are regarded as cellular mechanisms of apoptosis induction in ovarian cancer (Lin et al., 2013). However, unlike evidence reported by Lin et al. (2013), we observed the co-localization of p53 and COX-2 at nuclear level neither in the Caov-3 cell line nor in the HeLa and OV2008 cell lines. Interestingly, the combined treatment induced a reduction of p-p53(Ser15) in the nucleus of HeLa cells may be related to its export to cytoplasm, where it co-localized with COX-2, thereby suggesting a cooperative activity by these effectors in inducing apoptosis in such cervical cancer cells. However, we did not observe this effect in the other CC cell line, OV2008. Literature data reported that p53 may drive apoptosis in tumor cells by acting both at the nuclear level, by promoting the transcription of pro-apoptotic genes, such as Bax and Bak, and at the cytoplasmic level, by directly binding to anti-apoptotic mitochondrial proteins Bcl-2 and Bcl-XL, and resulting in neutralization of their inhibitory effects. By assessing possible changes in mitochondrial membrane potential, we found that in both OC and CC cell lines, the mitochondrial membrane depolarization is involved in both trabectedin and combined treatment-induced apoptosis. Collectively, these results support the hypothesis of a close relationship between activated p53 and COX-2 in apoptosis induction, but further investigations will be needed to better understand the molecular basis of their cooperation and role in apoptosis. Furthermore, we investigated the impact that trabectedin plus propranolol have on immune checkpoints expression PD1/PD-L1 in order to evaluate if this combined regimen could enhance the response to immunotherapy with anti-PD1/PD-L1. Neither drug modulated PD-L1. However, it did not mean that the combination had no effect on the response to immunotherapy because there are data in the literature reporting that trabectedin does not increase the expression of PD-L1 in *in vitro* OC models but only in *in vivo* (Guo et al., 2015) and thus, the absence of modulation in *in vitro* models is not indicative of a lack of efficacy of anti-PD1 (Guo et al., 2015). Trabectedin and/or propranolol reduced PD1 expression in OC cells, while in CC ones, we observed opposite effects. Thus, we are convinced that only an in-depth analysis of the direct interaction of these two drugs with anti-PD1 can give a real idea of their immunomodulatory activity, and we intend to deepen it in the future.

In conclusion, our study provided strong evidence that trabectedin is effective in inhibiting the proliferation of cervical cancer, and the combination strategy with propranolol further increased its efficacy. Although the underlying mechanisms of drug combination effectiveness need further investigation, the present study highlighted that trabectedin and, to a higher extent, the combined treatment hindered the DDR response pathway in the tested gynecological cancer models and offset resistance mechanisms elicited by β-ARs activation, promoting apoptosis. Overall, the results prompt further investigations on the use of trabectedin either alone or in combination therapy with propranolol in gynecological cancer types, mainly in cervical cancer.

## Data Availability

The raw data supporting the conclusion of this article will be made available by the authors, without undue reservation.
