# Supplementary material for: Cervical cancer benefits from trabectedin combination with the β-blocker propranolol: in vitro and ex vivo evaluations in patient-derived organoids
Source: Front Cell Dev Biol. 2023 Jun 13;11:1178316. doi: 10.3389/fcell.2023.1178316 (PMC10294430; doi:10.3389/fcell.2023.1178316)

Supplementary Material

Cervical cancer benefits from Trabectedin combination with the β-blocker Propranolol: in vitro and ex vivo evaluations in patients-derived organoids

Roberta Di Fonte[1], Sabino Strippoli[1], Marianna Garofoli[1], Gennaro Cormio[1], Simona Serratì[1], Vera Loizzi[1], Rossella Fasano[1], Francesca Arezzo[2], Mariateresa Volpicella[3], Afshin Derakhshani[4], Michele Guida[1], Letizia Porcelli[1+] and Amalia Azzariti[1,+,*]

[1] IRCCS Istituto Tumori "Giovanni Paolo II", 70124 Bari, Italy

[2] Unit of Obstetrics and Gynecology, Department of interdisciplinary Medicine, Policlinico Hospital, "Aldo Moro" University of Bari, 70124 Bari, Italy

[3] Department of Biosciences, Biotechnologies and Biopharmaceutics, University of Bari, 70126 Bari, Italy

[4] Department of Microbiology, Immunology & Infectious Diseases, Cumming School of Medicine, University of Calgary, Calgary, Canada

[+] These authors contributed equally as senior authors

*** Correspondence:**
Corresponding Author: Amalia Azzariti, a.azzariti@oncologico.bari.it; phone: +390805555986

Supplementary Figures

Supplementary Figure 1: Cell target modulation by treatment(s) in OC and CC cell lines, in presence of 10 µM NE. (A) Immunoblots conducted in OV2008 cell line after 24 h of treatment(s). (B) Histogram plots showing COX-2 mRNA expression level (vs GAPDH mRNA expression level) measured after treatment(s) in HeLa, OV2008 and Caov-3 cell lines.

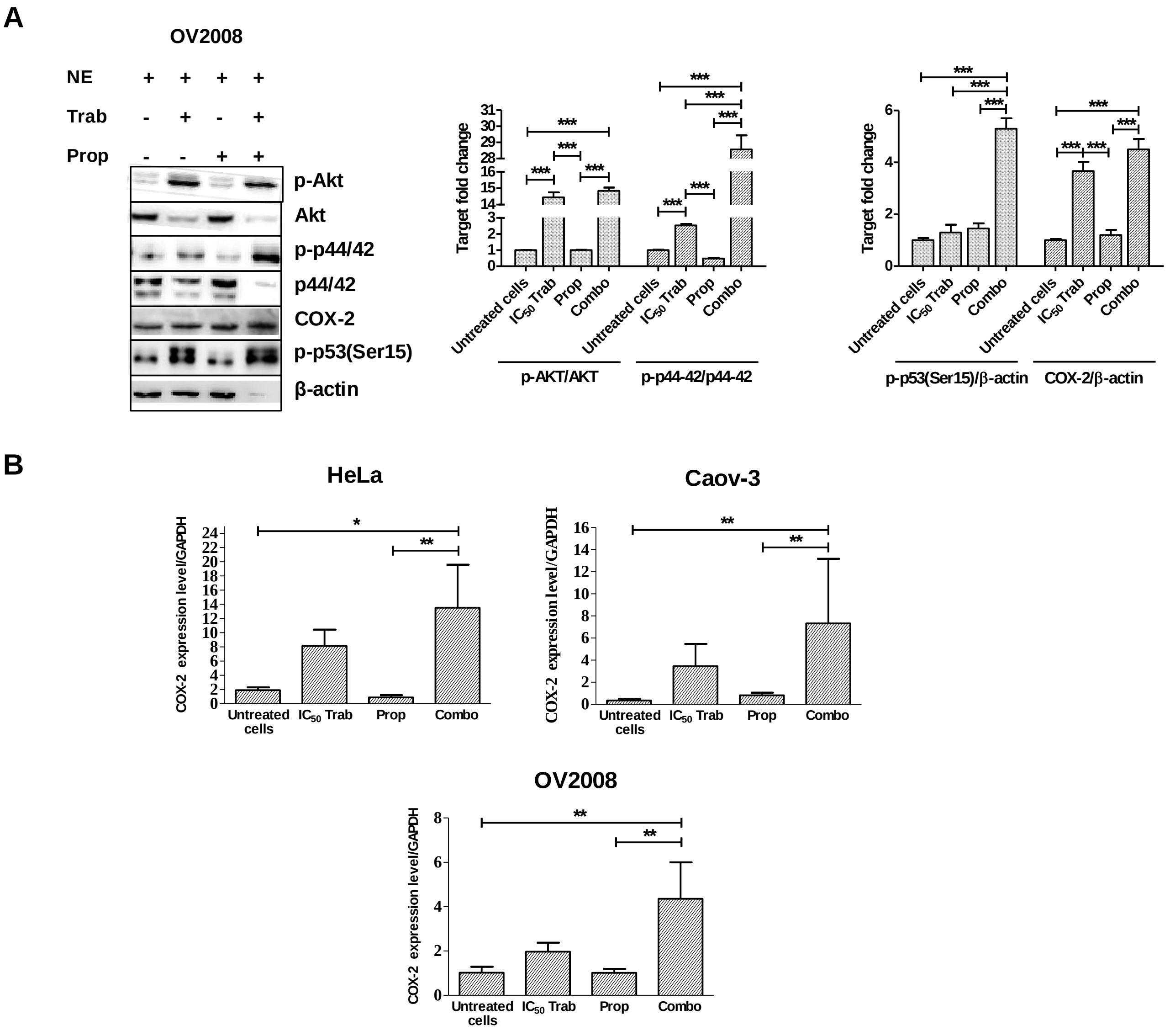

Supplementary Figure 2: IF evaluation of p-p53(Ser15) and COX-2 in OV2008 cell line in untreated and treated cells. Representative IF images showing p-p53(Ser15) and COX-2 localization in OV2008 cell lines after 24 h exposure to IC50 Trabectedin and Propranolol alone or in combination, in presence of 10 µM NE.

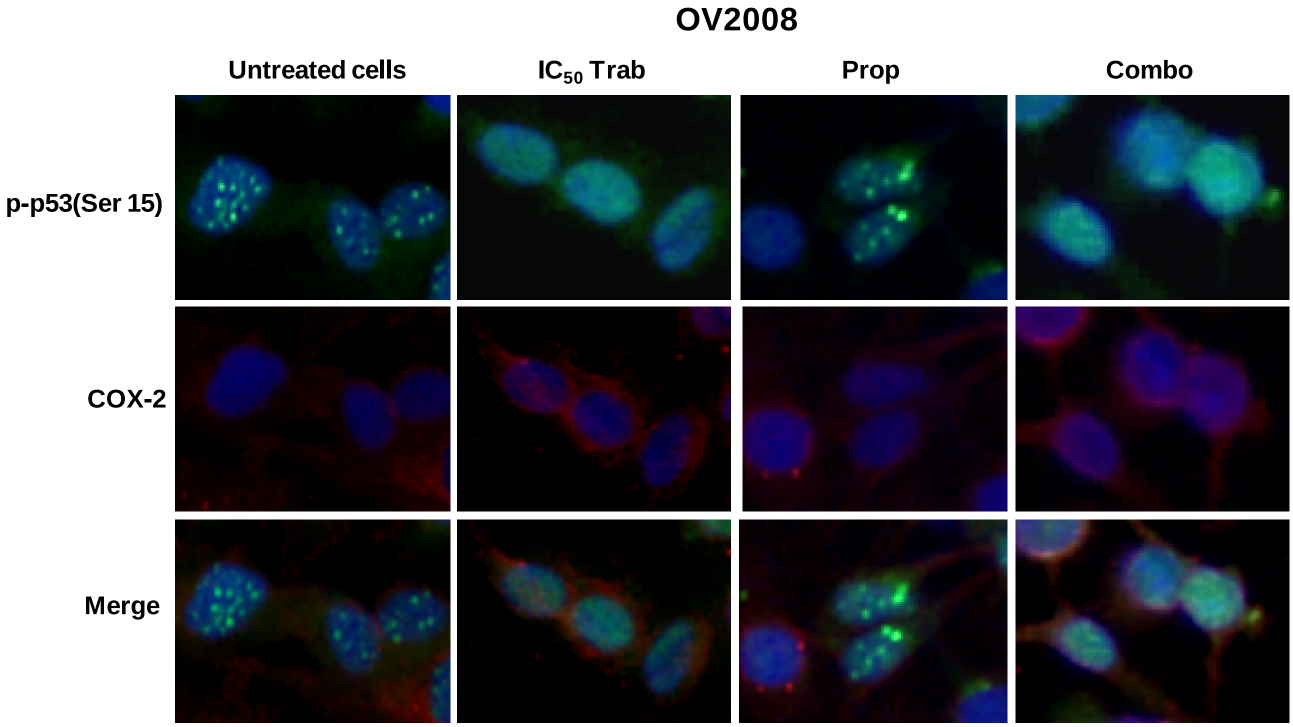

Supplementary Figure 3: PD1 and PD-L1 expression modulation by treatment(s) in OC and CC cell line. Histogram plots showing the expression (%) of PD-L1 (A) and PD1 (B) in OC and CC cell lines, performed by FCM in untreated and 24 h-treated cells with Trabectedin and Propranolol alone and in combination, under stress stimuli with 10 µM NE; the results are reported as mean±SD of three independent experiments. **p<0.01, ***p<0.001.

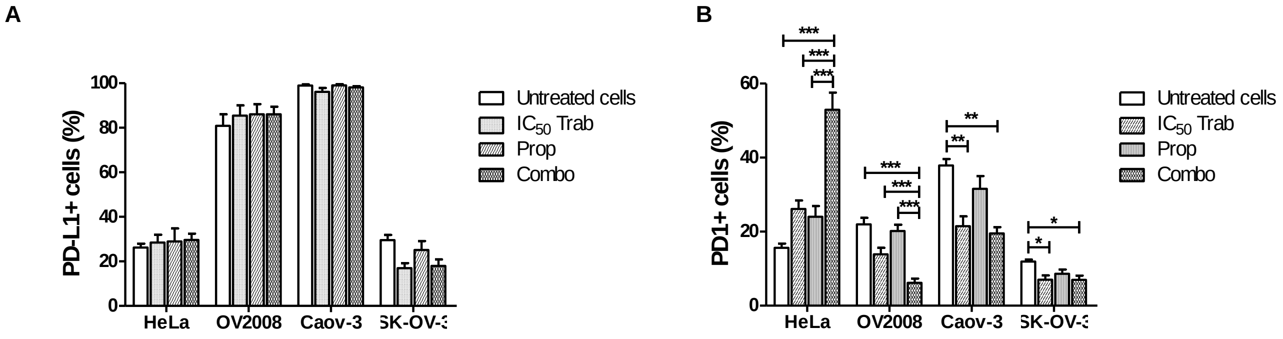

Supplement: Supplementary file 1 [file Image1.pdf]
